# Reconstruction of osteosarcoma of the proximal tibia using bone on polyethylene hemiarthroplasty knee joint system: A case report

**DOI:** 10.1016/j.ijscr.2020.05.013

**Published:** 2020-05-21

**Authors:** Yogi Prabowo, Muhammad Rizqi Adhi Primaputra, Evelina Kodrat

**Affiliations:** a Department of Orthopaedic & Traumatology, Cipto Mangunkusumo National Central Hospital and Faculty of Medicine, Universitas Indonesia, Jalan Diponegoro No. 71, Central Jakarta, Jakarta 10430, Indonesia; b Department of Anatomical Pathology, Cipto Mangunkusumo National Central Hospital and Faculty of Medicine, Universitas Indonesia, Jalan Diponegoro No. 71, Central Jakarta, Jakarta 10430, Indonesia

**Keywords:** Osteosarcoma, Reconstruction, Polyethylene, Hemiarthroplasty

## Abstract

**Introduction:**

Current treatment in treating patient with osteosarcoma is combination of chemotherapy and surgery, either limb-sparing or limb-ablation surgery. One challenge in limb-sparing technique in children is how to deal with the remaining growth of the bone. We created a limb-sparing reconstruction technique that can be fitted in many types of hospital and have a good functional outcome.

**Case presentation:**

We reported a case of 13-years-old female with left knee pain since 8 months prior hospital admission with previous treatment to a bone setter and open biopsy at previous hospital. Histopathological findings demonstrated osteosarcoma. The patient had a neoadjuvant chemotherapy and followed with limb-sparing surgery. We performed wide excision of proximal tibia and reconstruction using a bone on polyethylene hemiarthroplasty system of the knee joint. Patient then continued on adjuvant chemotherapy. Later on, local recurrence occurred and we performed excision again. Within one year, the patient could walk full weight bearing, perform daily activities with no limitation and no pain, and she had MSTS score of 21.

**Discussion:**

Limb-sparing surgery remains a challenging procedure in skeletally immature patients. This bone on polyethylene technique would be functional due to high adaptability in paediatric patients in order to decrease the number of surgeries until the final goal of limb equalization.

**Conclusion:**

Bone on polyethylene hemiarthroplasty system enables good and reliable functional outcome while maintaining the knee joint for daily activity. It can be chosen as one of viable options in treating osteosarcoma around the knee joint in children.

## Highlights

• Limb-sparing surgery remains a challenging procedure in skeletally immature patients.
• This bone on polyethylene technique would be functional due to high adaptability in paediatric patients in order to decrease the number of surgeries until the final goal of limb equalization.
• Such techinique enables good and reliable functional outcome while maintaining the knee joint for daily activity.

## 1 Introduction

Osteosarcoma is the most common primary malignant bone tumor in children [1]. The most common site is distal femur, followed by proximal tibia [2], [3]. Current treatment in treating patient with osteosarcoma is combination of neoadjuvant and adjuvant chemotherapy and surgery, either limb-sparing or limb-ablation surgery. One of the challenge in limb-sparing technique in children is how to deal with remaining growth of the bone [4], [5]. One of the techniques for limb-sparing reconstruction around the knee to solve the problem is endoprostheses replacement using modular type [4], [6]. However, this modular endoprostheses type cannot be done in our country due to its high cost. Therefore we created a limb-sparing reconstruction technique that can be fitted in many types of hospital and have a good functional outcome. This work has been reported in line with the SCARE criteria [7].

## 2 Case presentation

A 13-years-old female presented with pain on her left knee since 8 months ago. The pain was continuous and got worse at night. The patient went to a traditional masseuse and was given topical herbal ointment. Three months later, a lump appeared at her knee at a size of table tennis ball and getting bigger. The patient then went to a general surgeon, and histopathological examination demonstrated bone tumor. She was subsequently referred to our hospital.

**Fig. 1:**
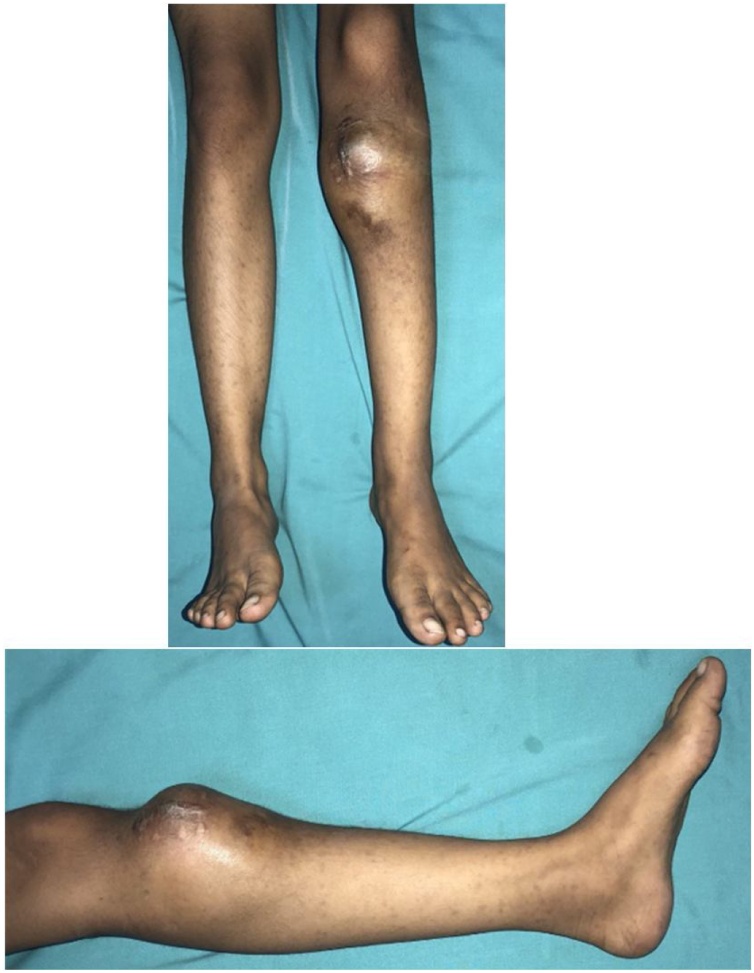
Clinical Picture After Chemotherapy.

Physical examination demonstrated a lump on the anterior part of proximal tibia with previous surgical biopsy scar and venectation. The lump had hard consistency with ill-defined border, smooth surface, immobile, and pain (visual analogue scale 2-3). Size circumferential of the leg was 33 cm compared to 27 cm on the contralateral. Neurovascular was good. The range of movement of both of the knee and ankle was normal (Fig. 1).

**Fig. 2:**
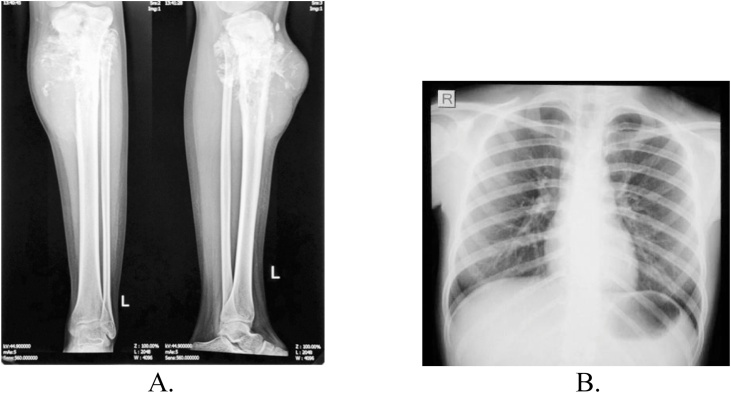
A. Cruris xray showed a mixed lytic and blastic lesion. B. Thorax xray showed no sign of metastasis.

**Fig. 3:**
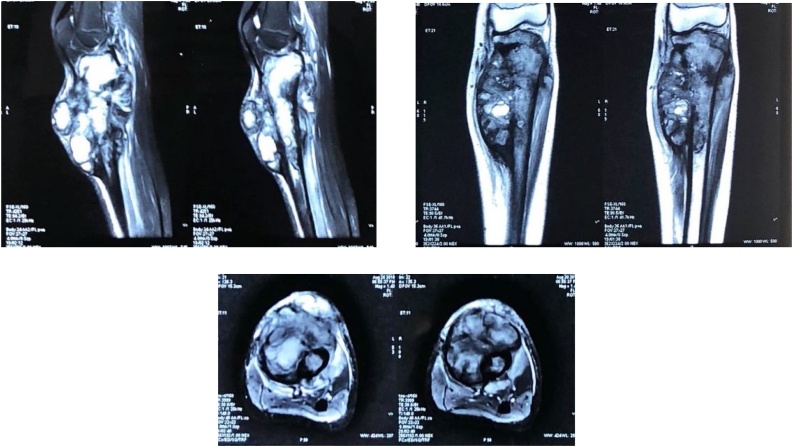
MRI showed a heterogenous mass in epiphysis extending to diaphysis of tibia, 16 cm from tibiofemoral joint, without neurovascular bundle involvement.

Plain radiography suggested mixed lytic and blastic bony lesion in metaphyseal area of proximal tibia, with ill-defined margin, wide transitional zone, periosteal reaction, matrix osteoid and also soft tissue involvement (Fig. 2a). Plain radiography of chest showed no sign of pulmonary metastasis (Fig. 2b). Contrast-enhanced magnetic resonance imaging (MRI) showed heterogeneous mass and no neurovascular bundle involvement (Fig. 3). Laboratory examination demonstrated increased erythrocyte sediment rate 47 mm/hour (N: < 15), and lactate dehydrogenase 717 U/L (N: < 480).

We did a review slide of the previous biopsy sample by the pathology of anatomy in our hospital. The result came out an osteosarcoma with osteoblastic cells. Then the case was brought to clinicopathological conference (CPC – A board consisting of experts from Orthopaedics Surgeon, Radiology, and Pathology Anatomy Department) – and the patient underwent chemotherapy neoadjuvant followed with surgery by limb salvage surgery procedure, wide excision and reconstruction with hemiarthroplasty system. The patient underwent chemotherapy neoadjuvant for 4 cycles weeks with regiments Cisplatin and Doxorubicin. After that, patient underwent a limb salvage surgery.

**Fig. 4:**
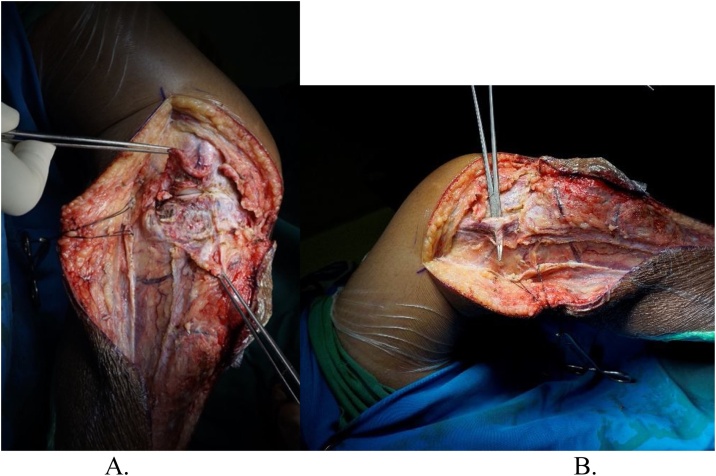
A. Patellar tendon preservation. B. Medial collateral ligament preservation.

**Fig. 5:**
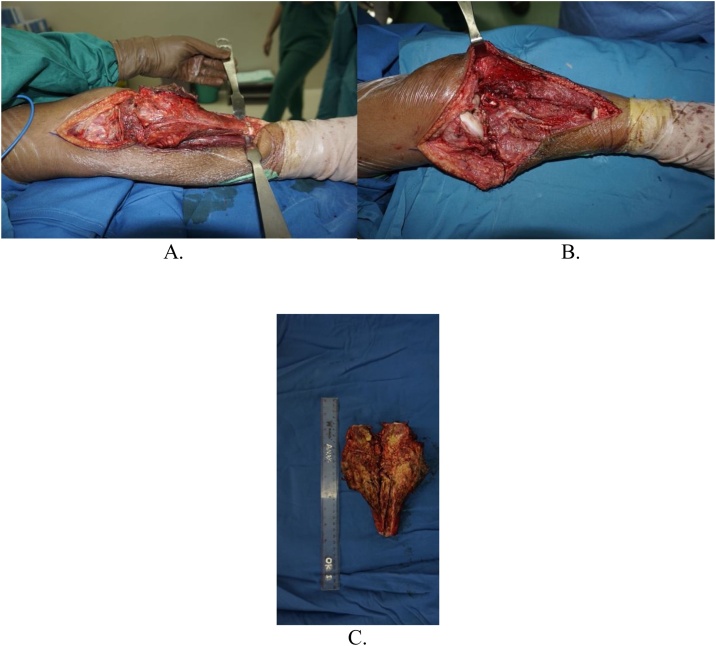
A. Tumor exposed. B. Bone defect 19 cm after tumor removal. C. Gross pathology of the tumor.

**Fig. 6:**
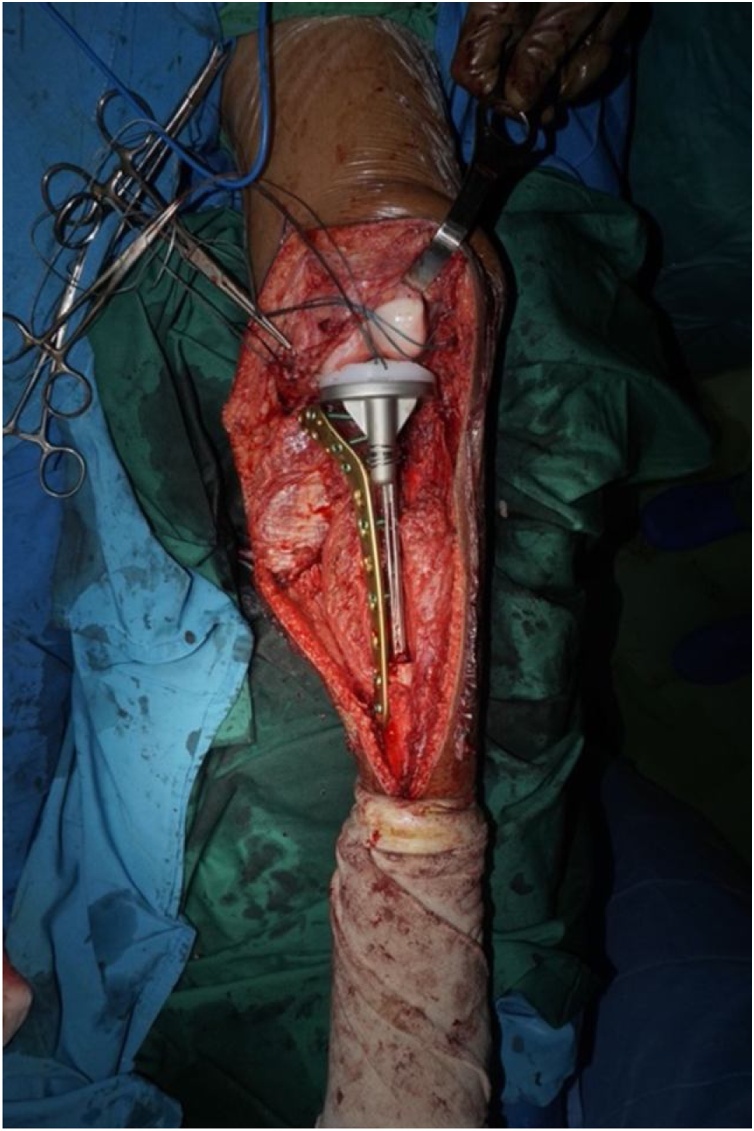
Construction of The Implant. We drilled the insert component of the tibial to make a hole later for suturing the remaining ACL and PCL. We added K-nail below the stem of base plate tibia and combined it with proximal tibial plate using cerclage wire.

**Fig. 7:**
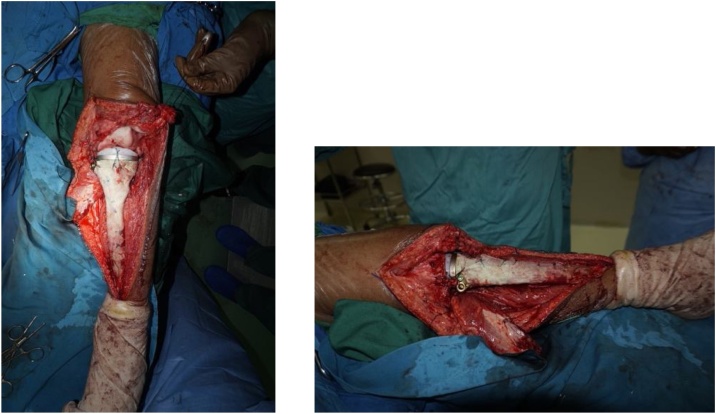
Post patellar tendon and MCL reconstruction. We sutured the remaining patellar tendon, MCL, ACL and PCL using polyester suture to the Mesh.

**Fig. 8:**
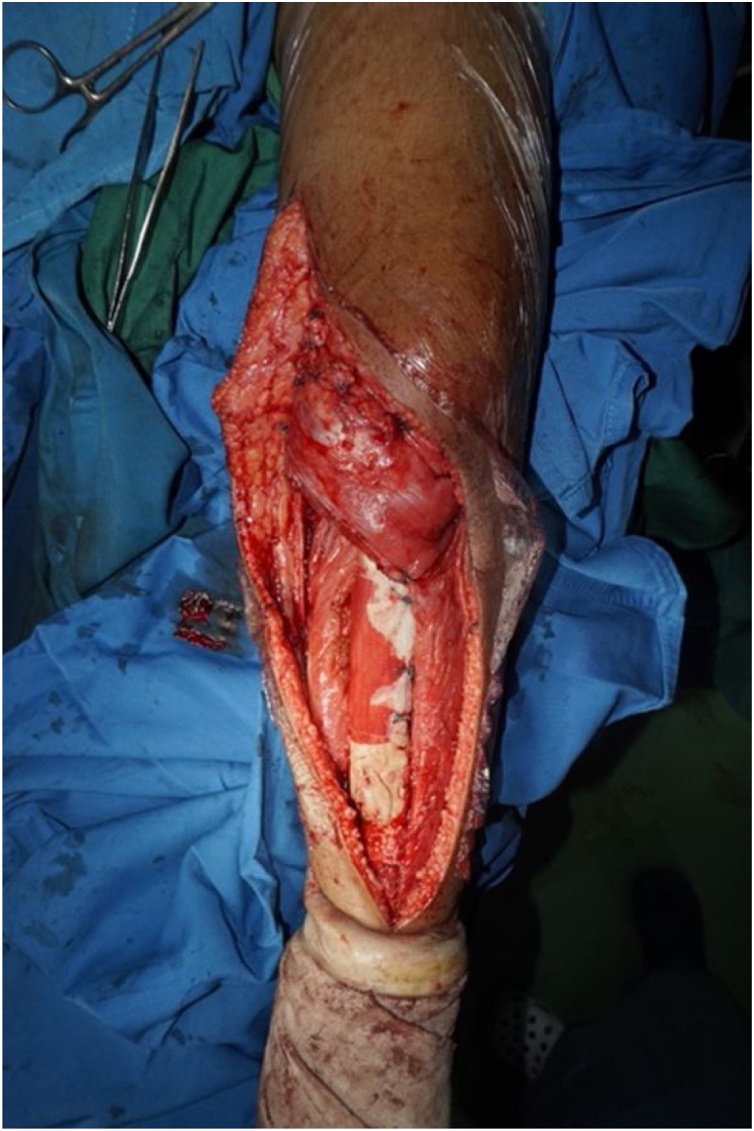
Final result with medial gastrocnemius flap.

**Fig. 9:**
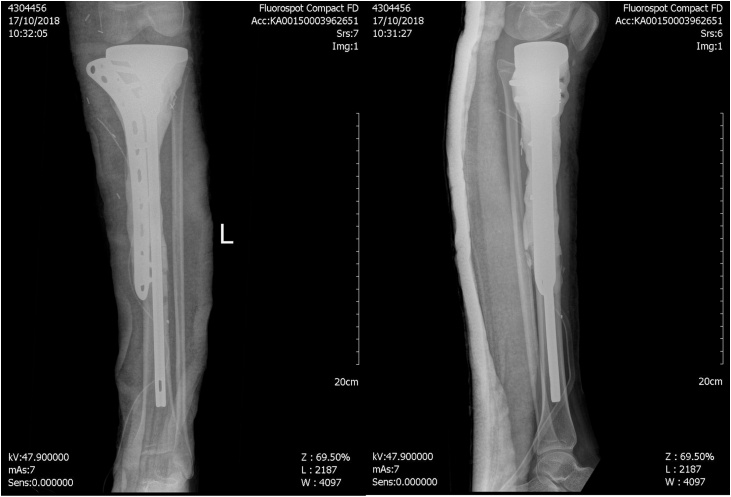
Post operative xray.

**Fig. 10:**
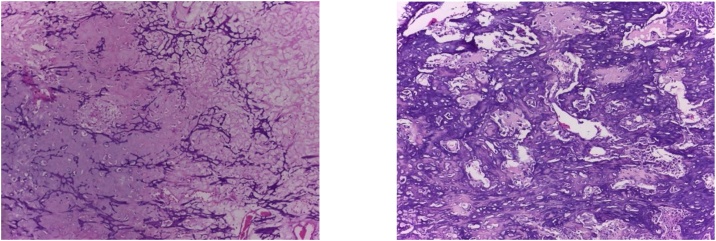
Histopathology feature showed osteosarcoma post neoadjuvant chemotherapy with 75% necrosis of the tumor cell consisted with HUVOS 2. (H&E, 400×).

Intraoperatively, we managed to perform wide excision, while preserving some part of patellar tendon, medial collateral ligament, and cruciate ligament (Fig. 4). We excised the tumor and then we measured the bone defect is 19 cm from joint line (Fig. 5). Then we reconstructed a hemiarthroplasty system to fill in the defect. We used a tibial component and insert of the primary total knee replacement implant and we connected to the distal using a Kuntscher Nail and a proximal tibial locking plate. We put the nail intramedullary to distal remaining bone (Fig. 6). We fixated all the implants with bone cement and we reconstructed the soft tissue using a mesh in anterior side of implants. We reconstructed the cruciate ligament through the insert and tied to the mesh and also we tied all the medial collateral ligament and the remaining patellar tendon to the mesh (Fig. 7). We added medial gastrocnemius flap to add coverage of soft tissue covering the implants (Fig. 8). We put a posterior splint directly after operation (Fig. 9). The patient routinely controlled to our outpatient clinic every month. After 6 weeks, we removed the splint and patient started range of motion 6 weeks after and partial weight bearing using bilateral crutches. Subesquently, we confirmed the histopathology result for HUVOS result (Fig. 10) and continued with chemotherapy adjuvant for another 5 cycles with regiments of iphosphamide, cisplatin and adriamycin.

**Fig. 11:**
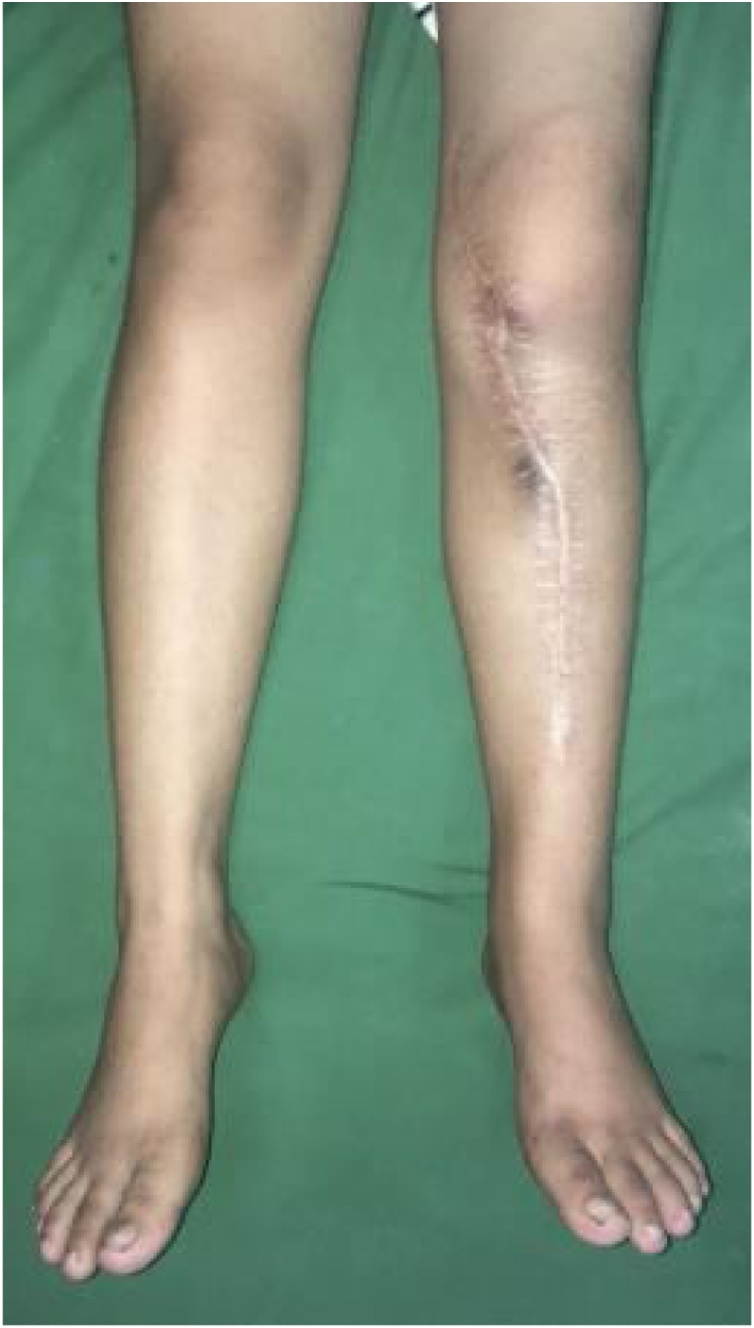
Clinical picture showed a lump on previous scar.

**Fig. 12:**
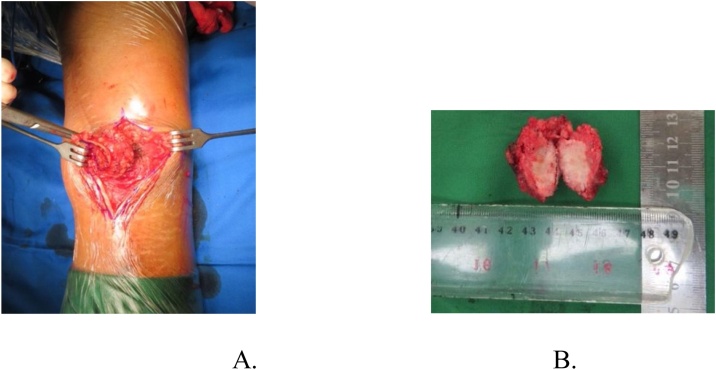
A. Tumor exposed. B. Gross pathology of the tumor.

**Fig. 13:**
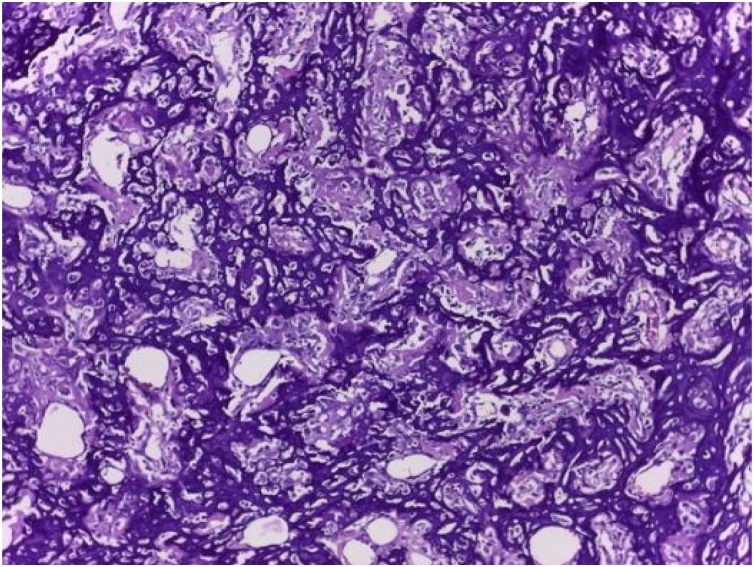
Histopathology result showed the same appearance was observed at recurrence site (H&E, 100×).

Eight months later, lump re-appeared at the same site of previous lesion with pain. The lump size was 2 × 2 × 2 cm with hard consistency and pain visual analogue scale 2-3 (Fig. 11). No venectation, no sinus or wound appeared. X-ray confirmed that position of the implant still good with no bony lesion. Then, we did excision of the mass and it came out the result was osteosarcoma (Fig. 12, Fig. 13).

**Fig. 14:**
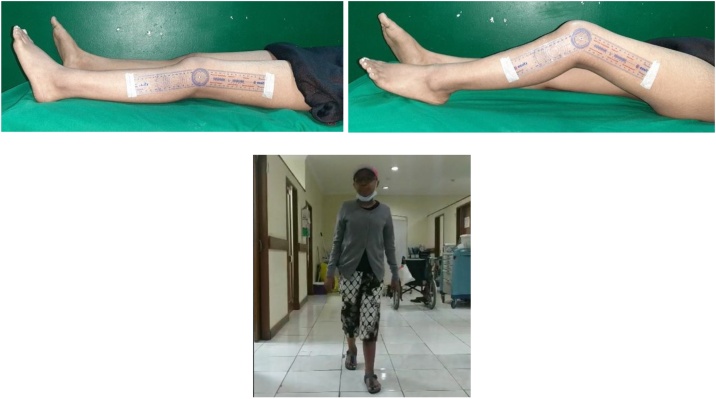
Clinical outcome after 1 year of surgery. Patient can walk without any additional aid and no pain. Flexion and extension 10–50 degrees.

On 1 year of follow-up, the patient could walk with full weight bearing without any additional aid. She complained no pain, and she could do her daily activity well. The leg length discrepancy was approximately 1 cm, her knee flexion-extension range of motion was 10 to 50 degrees within stable varus valgus test, motoric strength 4, and the MSTS score was 21 (Fig. 14).

## 3 Discussion

Osteosarcoma is a malignant tumor which arises from a mesenchymal stem cell precursor that produces immature woven bone (osteoid) [1], [5]. It is the most common paediatric bone malignancy [6]. The peak incidence of this malignancy is between the age of 10 and 20 years of age, and the annual incidence is approximately 2 to 3 per 106,000 individuals [5]. The most frequent sites of such tumor include distal femur, proximal tibia and proximal humerus [2], [3].

In the past, osteosarcoma was treated by amputation solely. Nowadays, neoadjuvant preoperative and adjuvant chemotherapy regimens exist, and they allow safe limb-sparing resections, thereby improving survival rates [1], [2], [3], [5], [8]. Neoadjuvant chemotherapy has several goals: 1) reducing tumor size, 2) clearing micro-metastasis, and 3) facilitating better margination of the primary tumor thus allowing limb salvage surgery to be performed if possible [1]. The total duration of the treatment ranged from 6 to 10 months; this depends on chemotherapy response [1], [6]. With the recent availability of multimodal treatment combining chemotherapy, and surgical techniques, 70–85% of malignant tumors could be efficiently treated with limb salvage. With current modalities, the 5-year overall survival and event-free survival rates achieved was about 70% and 58%, respectively. However, those who present with metastatic disease still have unfavourable outcomes, with overall 5-year survival of approximately 34% [2], [5], [6], [8], [9].

Limb-salvage surgery provides good oncological and functional outcomes, as well as satisfactory psychological results [1]. Despite numerous reports regarding the surgical techniques (e.g. allografts and arthrodesis), the treatment options for reconstructing bone and soft-tissue defects after resection remain a serious challenge for orthopaedic surgeons, particularly in skeletally immature patients [4], [5], [9], [10], [11]. As the epiphysis of both sides of the knee joint account for more than two-thirds of longitudinal lower limb growth, resection of osteosarcoma around knee joints in skeletally immature patients presents a concern regarding limb-length discrepancy (LLD) [4], [5], [9], [10], [11], [12].

With recent advances in prosthesis design, modular cemented endoprostheses are abundantly used to reconstruct distal femur and proximal tibia defects after bone tumor resection. These endoprostheses are frequently used due to their low incidence of aseptic loosening; moreover, they provide better functional outcomes [1], [5], [6], [9], [13].

The proximal tibia is the second most common site of osteosarcoma after distal femur. This procedure is challenging due to various factors. Often, complications and loss of function following resection of the extensor mechanism occur [14], [15]. Numerous methods are available to improve extensor mechanism; these include direct reattachment of the patellar tendon to the prosthesis and attachment of the patellar tendon to the transposed gastrocnemius muscle flap [14], [15]. Medial gastrocnemius muscle flap also shown to decrease the risk of infection and promote good healing [14], [15], [16]. Allografts were used in order to decrease the risk of loosening, synovitis, and trauma [16].

To avoid the high cost of modular endoprosthesis and preserve knee joint preservation in children, we designed a combination of arthroplasty and internal fixation to overcome the defect after tumor resection. We combined the tibial component of total knee arthroplasty, internal fixation devices, kunstcher nail, proximal tibia locking plate, and cerclage wire, and bone cement. This technique bone on polyethylene system, enhanced with bone cement, promises low risk of loosening. We preserved the patellar tendon and attached it using mesh to maintain the previous extensor mechanism of knee joint. The anterior and posterior cruciate ligament and medial collateral ligament were preserved to improve stability of the knee joint. Although there was some little instability on valgus stress test intraoperative, but later on the follow up instability was gone due to its fibrotic tissue. The patient also did not complain any symptoms and could walk full weight bearing without any additional aid. Finally, in order to decrease the risk of infection, medial gastrocnemius rotational flap was used. This technique, bone on polyethylene, was created to make an alternative to modular endoprosthesis. Such procedure would be functional due to high adaptability in paediatric patients. Furthermore, it may decrease the number of surgeries until the final goal of limb equalization and adult-type tumor prosthesis implantation.

Complications of endoprostheses include infection, aseptic loosening, soft tissue failure and fracture of the prostheses or bone. Local recurrence and aseptic loosening often lead to secondary amputation and revision, which are a major concern for prosthetic failure. Therefore, negative margins should be achieved in tumor resection and chemotherapy in order to prevent local recurrence. Complications resulting from local recurrence occurred in 9.3% of patients. Both local recurrence and deep infection are the two main causes of secondary amputation and revision. In most cases, local recurrence occurred within three years after surgical procedure [1], [5], [6], [9], [13].

## 4 Conclusion

Combination chemotherapy and limb-salvage surgery in osteosarcoma gives good survival rate outcome. This bone on polyethylene hemiarthroplasty system enables good and reliable functional outcome while maintaining the knee joint for daily activity. It can be chosen as one of viable options in treating osteosarcoma around the knee joint for children, besides doing a modular endoprosthesis. It costs less than modular endoprosthesis and can be done in many types of hospital. In that way, children patients with osteosarcoma can be treated early and have a better survival rate.

## Declaration of Competing Interest

None.

## Funding

None declared.

## Ethical approval

Ethical approval has been received from Cipto Mangunkusumo Hospital, Jakarta, Indonesia.

## Consent

Written informed consent was obtained from the patient’s parents for publication of this case report and accompanying images. A copy of the written consent is available for review by the Editor-in-Chief of this journal on request.

## Author contribution

Yogi Prabowo performing the procedure, study concept, data collection.

Muhammad Rizqi Adhi Primaputra: data collection, writing the paper.

Evelina Kodrat: data collection.

## Registration of research studies

This study has been registered at researchregistry.com (UIN: researchregistry5169).

## Guarantor

Yogi Prabowo.

## Provenance and peer review

Editorially reviewed, not externally peer-reviewed.
